# Investigation of the quality of life and influencing factors among perimenopausal women

**DOI:** 10.1007/s00404-025-08116-1

**Published:** 2025-07-23

**Authors:** Xiangrong Liu, Xiumin Zhang, Dianhui Wang, Jing Zhou, Yu Li

**Affiliations:** 1 Department of Gynecology, The Second Hospital of Shandong University, Jinan, 250033 Shandong China; 2 Department of Endocrinology and Metabolism, The Second Hospital of Shandong University, Jinan, 250033 Shandong China

**Keywords:** Perimenopause, Quality of life, Sleep, Motor activity, Anxiety, Depression, Cross-sectional studies

## Abstract

**Purpose:**

Perimenopause, the transitional phase preceding menopause, is characterized by hormonal fluctuations causing physical, psychological, and sexual symptoms collectively known as perimenopausal syndrome. These symptoms can substantially impair quality of life (QoL), particularly in the context of an aging global population. This study aimed to evaluate the QoL among perimenopausal women and identify factors influencing it.

**Methods:**

This cross-sectional study was conducted at the Second Hospital of Shandong University from January 2022 to December 2023. A total of 387 perimenopausal women were selected by convenience sampling. Data were collected using MENQOL, PSQI, IPAQ-L, SAS, and SDS scales. Statistical analyses included Pearson correlation, one-way ANOVA, rank-sum tests, and stepwise multivariate linear regression (P < 0.05).

**Results:**

The mean MENQOL score was 51.59 ± 30.15, indicating moderate overall quality-of-life impairment. The mean PSQI score was 9.97 ± 5.89; 70.54% of participants had poor sleep quality. Anxiety symptoms were present in 58.9% of participants, and depressive symptoms in 68.7%. Leisure-time physical activity was low (median: 396 MET-min/week), while sedentary behavior was high (mean: 1598 ± 903 min/week). Correlation analysis showed total MENQOL scores to be positively associated with PSQI (r = 0.579, P < 0.01), SAS (r = 0.096, P > 0.05), and SDS scores (r = 0.059, P > 0.05), and negatively associated with sleep duration (r = − 0.380, P < 0.01). Work-related physical activity was also positively associated with MENQOL scores (r = 0.144, P < 0.01). Multivariate regression identified the following independent predictors of poorer quality of life: poorer sleep quality, shorter sleep duration, higher work-related physical activity, presence of anxiety or depression, older age, and comorbid conditions.

**Conclusion:**

The QoL of perimenopausal women is affected by a multifactorial interplay of lifestyle behaviors, psychological status, and chronic health conditions, highlighting the importance of early screening and tailored interventions, particularly those addressing sleep disturbances, mental health, and appropriate physical activity.

## What does this study add to the clinical work

**Table:** 

The quality of life in perimenopausal women is influenced by multiple factors. This study comprehensively assesses the current status of quality of life in perimenopausal women and explores its associated influencing factors. The findings provide a theoretical basis for developing effective prevention and intervention strategies to alleviate perimenopausal syndrome, offering both theoretical support and practical guidance to improve the quality of life in this population.	

## Introduction

Perimenopause is the natural physiological stage marking the transition to menopause, during which ovarian function declines and age-related metabolic changes occur [1]. According to the North American Menopause Society, this period encompasses the years leading up to—and the year following—the final menstrual period, typically between ages 40 and 60 [2]. Hormonal fluctuations in this phase give rise to a range of symptoms collectively known as perimenopausal syndrome [3].

Perimenopausal symptoms include vasomotor disturbances, menstrual irregularities, and increased cardiovascular risk, as well as genitourinary complaints such as vaginal dryness and discomfort during sexual activity [4, 5]. In addition, many women experience sleep disturbances and mood fluctuations, which affect both physical health and psychological well-being, ultimately reducing their overall quality of life [6]. Epidemiological studies suggest that factors such as age at menopause, psychological status, and lifestyle habits influence quality of life during the perimenopausal period [7].

The burden of perimenopausal health issues is projected to grow as the global population ages. The World Health Organization estimates that by 2030, there will be 1.2 billion menopausal women worldwide, with approximately 280 million in China alone [8]. Epidemiological data indicate that over 80% of women in Europe and North America experience menopausal symptoms, and more than 70% of Chinese women report varying degrees of perimenopausal discomfort [9]. Additionally, sleep disturbances are highly prevalent among perimenopausal women and are strongly associated with reduced quality of life (QoL) [10]. Poor sleep quality not only increases fatigue and irritability but also exacerbates vasomotor and psychological symptoms, leading to greater overall distress [11]. However, inconsistencies exist regarding the reported prevalence and severity of sleep problems, with some studies suggesting they are transient and others indicating a persistent, long-term impact [12].

Mental health plays a central role in QoL during perimenopause. Anxiety and depressive symptoms are frequently reported and often co-occur with somatic complaints [13]. Higher levels of anxiety and depression have been independently associated with worse QoL scores across physical, psychological, and sexual domains [14]. Nevertheless, some studies report only modest associations, suggesting that individual coping styles and social support may modify these effects [15]. Physical activity is another modifiable factor linked to improved QoL. Regular exercise can alleviate mood symptoms, reduce sleep disturbances, and improve cardiovascular and musculoskeletal health in menopausal women [16]. Despite these benefits, findings remain inconsistent: some studies indicate that occupational physical activity may not confer the same advantages as leisure-time activity, and excessive physical workload may even worsen menopausal symptoms [16].

Although vasomotor disturbances and genitourinary complaints are well documented [17], relatively few studies have comprehensively assessed the combined impact of sleep, psychological status, and physical activity on QoL during perimenopause, particularly in Chinese women. Cultural stigma surrounding mental and sexual health further contributes to underreporting and delays in seeking care, with only 18.9–25% of women pursuing medical consultation for such issues [18]. This gap in health-seeking behavior leaves many symptoms untreated, exacerbating both psychological and physical distress and increasing the burden on families and the healthcare system.

Despite the high prevalence of perimenopausal symptoms and their impact on well-being, QoL among Chinese perimenopausal women remains underexplored. Previous studies have primarily focused on isolated symptoms or limited regional samples, with insufficient attention to the broader range of contributing factors, including sleep, mental health, and physical activity [19, 20].

This study hypothesizes that perimenopausal women’s quality of life is significantly influenced by sleep quality, psychological status, physical activity levels, and comorbid health conditions. Accordingly, our objective was to assess current QoL in perimenopausal women and identify key determinants through a standardized, multidimensional evaluation. These findings will inform clinical strategies for early screening and targeted interventions to enhance health outcomes and overall quality of life in this population.

## Methods

### Study participants

This descriptive cross‐sectional study used a convenience sampling method. We recruited 387 women who visited the combined menopause–endocrinology outpatient clinic at the Second Hospital of Shandong University between January 2022 and December 2023. These women came primarily for routine health examinations or general menopausal counseling rather than treatment of specific pathologies, reducing selection bias and enhancing the applicability of our findings to the broader perimenopausal population.

Participants were eligible for inclusion if they: (1) were women aged 40–60 years entering the perimenopausal period according to the STRAW+10 criteria; (2) attended the combined menopause–endocrinology outpatient clinic for routine consultation or health assessment; (3) were capable of independently completing the survey; (4) voluntarily agreed to participate and provided informed consent; and (5) had no history of pregnancy, abnormal vaginal bleeding, hysterectomy, chemotherapy, or major physical or psychiatric illness at the time of enrollment. Participants were excluded if, after enrollment, any of the following occurred: (1) failure to complete the survey or missing critical data; (2) subsequent identification of a diagnosis inconsistent with the STRAW+10 criteria (e.g., premature ovarian insufficiency, polycystic ovary syndrome); (3) cognitive or intellectual difficulties discovered during data collection that compromised data reliability; or (4) withdrawal of participation or refusal by family members during the course of the study.

### Sample size determination

Sample size was calculated for multiple linear regression with 21 predictors, requiring at least 15 observations per variable (n = 315). Allowing for a 10% attrition rate due to invalid or incomplete responses raised the target to 350. To protect against further dropouts and data exclusions, an extra margin was added and 402 questionnaires were distributed. Six respondents were excluded for a history of surgical menopause and nine for incomplete data, yielding 387 valid responses (effective rate: 96.27%).

Ethical approval was granted by the institutional committee (Approval Nos. KYLL-2022-575 and KYLL2024LW032), and the study was supported by institutional seed funding (Project No. 2023HL05). All procedures complied with the Declaration of Helsinki [21], informed consent was obtained from each participant, and personal information was treated confidentially throughout.

### Data collection

All investigators underwent standardized training prior to data collection. Following informed consent, general demographic and clinical data were collected on-site. Participants completed structured questionnaires, and data were entered into a secure database using Epidata 3.1 with a double-entry procedure to ensure data accuracy.

### Instruments and measures

All questionnaires were administered in person by trained investigators who had more than 10 years of clinical outpatient experience and were proficient in patient communication. Prior to study implementation, a pilot test indicated that completing the full set of questionnaires required approximately 30 min. To minimize respondent burden and ensure data quality, the survey was conducted before the participants’ clinical consultation. Investigators provided one-on-one assistance and guided participants through the items in a quiet and private setting.

Participants with a high school education or above (79.85%) were able to complete the questionnaires independently. For those with lower educational attainment (20.15%), including those with less than a junior high school education, investigators explained each item clearly and assisted in filling out the questionnaires when necessary. No participants were illiterate. Additionally, to promote attentiveness and completeness, the attending specialist reviewed the completed questionnaires before the clinical interview.

#### General information questionnaire

This self-designed questionnaire was developed through literature review and expert discussion to collect demographic and reproductive health information, including age, residence, marital status, education level, employment status, economic status, height, weight, body mass index (BMI), number of deliveries and abortions, age at menarche, and history of hypertension, diabetes, coronary heart disease, and osteoporosis.

#### Menopause-specific quality of life questionnaire (MENQOL)

The MENQOL, developed by Hilditch et al. [22], assesses menopause-related quality of life across four domains: vasomotor symptoms (3 items; total score: 18), psychosocial status (7 items; 42), physical status (16 items; 96), and sexual function (3 items; 18). Each domain score is averaged, with lower scores indicating better quality of life.

The questionnaire demonstrated good psychometric properties. The internal consistency, as measured by Cronbach’s α, was 0.82 for the vasomotor domain, 0.81 for psychosocial, 0.87 for physical, and 0.89 for sexual domains. Discriminant validity coefficients were 0.40, 0.65, 0.69, and 0.58 for the vasomotor, psychosocial, physical, and sexual domains, respectively. Evaluative validity was reported as 0.28 for vasomotor, 0.55 for psychosocial, 0.60 for physical, and 0.54 for sexual domains [23, 24].

#### Pittsburgh sleep quality index (PSQI)

The PSQI, developed by Buysse et al. [25], evaluates subjective sleep quality over the past month. It consists of 19 self-rated and 5 observer-rated items, though only 18 items are used in scoring. These items form 7 components (each rated 0–3), resulting in a total score ranging from 0 to 21, with higher scores reflecting poorer sleep quality. In Chinese populations, the PSQI has demonstrated strong reliability and validity, with Cronbach’s α coefficients of 0.8420 (components) and 0.8519 (items), and a split-half reliability of 0.8661 [26]. A total score > 7 indicates poor sleep quality, with sensitivity and specificity of 98.3% and 90.3%, respectively.

#### International physical activity questionnaire-long form (IPAQ-L)

The IPAQ-L, translated into Chinese by Qu et al. [27], is a 27-item instrument used to assess physical activity over the past week. The questionnaire covers multiple domains, including work-related activities, household tasks, transportation, exercise, and leisure-time physical activity. Based on the intensity and frequency of activities, physical activity levels are categorized as high, moderate, or low [27].

Each activity type is assigned a metabolic equivalent of task (MET) value, and total activity is calculated by multiplying the MET score by frequency (days/week) and duration (min/day). Both the total score and subdomain scores are calculated in MET-min/week, with higher scores reflecting higher levels of physical activity. In validation studies, the IPAQ-L demonstrated good reliability, with a Pearson correlation coefficient of 0.743 for total energy expenditure.

#### Self-rating anxiety scale (SAS)

The SAS, developed by Zung in 1965 at Duke University School of Medicine [28], comprises 20 items designed to evaluate the presence and severity of anxiety symptoms. Each item is rated on a 4-point Likert scale ranging from 1 to 4, reflecting the frequency of symptom occurrence: “1 = a little of the time," “2 = some of the time," “3 = a good part of the time,” and “4 = most of the time.” The total raw score is obtained by summing the responses to all 20 items, yielding a possible range of 20–80. To derive the standard score, the raw score is multiplied by 1.25 and rounded to the nearest integer, with higher scores reflecting greater anxiety severity. According to Chinese normative data [29], a standard score > 50 is considered indicative of clinically significant anxiety. Anxiety severity is further classified as follows: < 50 = normal; 50–59 = mild anxiety; 60–69 = moderate anxiety; and ≥ 70 = severe anxiety.

#### Self-rating depression scale (SDS)

The SDS, developed by Zung [30], is a 20-item self-administered instrument designed to assess depressive symptoms. Each item is rated on a 4-point Likert scale reflecting the frequency of symptoms: “1 = a little of the time,” “2 = some of the time,” “3 = a good part of the time,” and “4 = most of the time.” The raw score is obtained by summing the responses to all 20 items, yielding a possible range from 20 to 80.

To calculate the standard score, the raw score is multiplied by 1.25, and the integer part is used for classification. Higher standard scores indicate greater severity of depressive symptoms. Based on Chinese normative data [31], depression severity is classified as follows: a standard score < 53 indicates normal status; 53–62 indicates mild depression; 63–72 indicates moderate depression; and a score > 72 corresponds to severe depression.

### Statistical analysis

All data were first entered into a database using Epidata 3.1 with a double-entry procedure to ensure accuracy. The finalized dataset was then exported to Microsoft Excel and analyzed using SPSS version 25.0 (IBM Corp., Armonk, NY, USA). Continuous variables were expressed as mean ± standard deviation for normally distributed data, and as median with interquartile range for non-normally distributed data. Categorical variables were presented as frequencies and percentages. Univariate analyses were performed using Pearson correlation, one-way analysis of variance (ANOVA), and rank-sum tests, as appropriate. For multivariate analysis, multiple linear regression with stepwise variable selection was employed to identify significant predictors. A two-sided P-value < 0.05 was considered statistically significant.

## Results

### General characteristics of perimenopausal women

The study included 387 perimenopausal women, predominantly aged 46–50 years (40.1%) and 40–45 years (30.7%), with a mean age of 47.94 ± 5.30 years. Most participants resided in urban areas (77.78%) and had attained at least a high school education (79.85%). The majority were married (93.8%) and covered by medical insurance (88.4%). Regarding health status, 7.49% reported a history of hypertension, 3.10% had diabetes, 1.29% had coronary heart disease, and 2.33% had osteoporosis (Table 1). These characteristics indicate a relatively educated, urban-dwelling population with a moderate burden of chronic disease.

**Table 1:** General characteristics of perimenopausal women (n = 387)

Variable	Group	Frequency (n)	Percentage (%)
Age (years)	40–45	119	30.70
	46–50	155	40.10
	51–55	85	22.00
	56–60	28	7.20
BMI (kg/m^2^)	< 18.5	8	2.10
	18.5–23.9	192	49.60
	24.0–27.9	156	40.30
	≥ 28	31	8.00
Ethnicity	Han	380	98.19
	Hui	6	1.55
	Others	1	0.26
Place of residence	Rural	38	9.82
	Town	48	12.40
	City	301	77.78
Education level	Primary school or below	23	5.94
	Junior high school	55	14.21
	High school or technical school	90	23.26
	Associate degree	63	16.28
	Bachelor’s degree	130	33.59
	Master’s degree or above	26	6.72
Occupation	Civil servant	5	1.29
	Professional/technical staff	139	35.92
	Clerical staff	73	18.86
	Enterprise manager	11	2.84
	Worker	40	10.34
	Farmer	24	6.20
	Self-employed	31	8.01
	Retired	35	9.04
	Unemployed	29	7.49
Marital status	Unmarried	5	1.29
	First marriage	363	93.80
	Remarried	15	3.88
	Divorced	4	1.03
Monthly household income per capita (yuan)	≤ 4999	111	28.68
	5000–9999	165	42.64
	10,000–19,999	83	21.45
	≥ 20,000	28	7.24
Payment method for medical expenses	Self-paid	42	10.90
	Provincial insurance	87	22.50
	Urban employee insurance	203	52.50
	Urban resident insurance	43	11.10
	Commercial medical insurance	9	2.30
	Others	3	0.80
Menopausal status	No	271	70.03
	Yes	116	29.97
Age of menarche (years)	≤ 12	91	23.51
	13–16	276	71.32
	≥ 17	20	5.17
Number of childbirths	0	13	3.36
	1	263	67.96
	2	106	27.39
	≥ 3	5	1.29
Number of abortions	0	120	31.01
	1	143	36.95
	2	89	23.00
	≥ 3	35	9.04
Hypertension	No	358	92.51
	Yes	29	7.49
Diabetes	No	375	96.90
	Yes	12	3.10
Coronary heart disease	No	382	98.71
	Yes	5	1.29
Osteoporosis	No	378	97.67
	Yes	9	2.33

*BMI* body mass index, *PSQI* Pittsburgh sleep quality index, *SAS* self-rating anxiety scale, *SDS* self-rating depression scale, *MENQOL* menopause-specific quality of life questionnaire, *MET* metabolic equivalent of task

### MENQOL and PSQI scores indicate moderate quality of life impairment and poor sleep quality

The average MENQOL score was 51.59 ± 30.15, reflecting a moderate level of quality of life impairment among the participants. Among the MENQOL domains, psychosocial functioning exhibited the greatest impact (mean score: 1.95 ± 1.34), followed by sexual life (2.37 ± 1.71), vasomotor symptoms (1.66 ± 1.64), and physical status (1.62 ± 1.03) (Table 2). The mean PSQI score was 9.97 ± 5.89, and 70.54% of participants scored > 7, indicating that poor sleep quality was highly prevalent in this cohort.

**Table 2:** Scores of perimenopausal quality of life, dimensional scores, and sleep quality (n = 387)

Item	Mean ($\overline{x}$) ± standard deviation (*s*)
Total MENQOL score	51.59 ± 30.15
Vasomotor symptoms mean score	1.66 ± 1.64
Psychosocial status mean score	1.95 ± 1.34
Physical status mean score	1.62 ± 1.03
Sexual life mean score	2.37 ± 1.71
PSQI	9.97 ± 5.89

*MENQOL* menopause-specific quality of life questionnaire, *PSQI* Pittsburgh sleep quality index

### Low leisure-time physiacal activity and high psychological distress among perimenopausal women

Participants reported a high average total physical activity level (5301.48 ± 4575.24 MET-min/week), with work-related activity contributing significantly. However, median levels of leisure-time physical activity were notably lower (396 MET-min/week), and sedentary time remained high (1598 ± 903 min/week). The average sleep duration was 2773 ± 555 min/week (approximately 6.6 h/day) (Table 3). Anxiety symptoms were present in 58.9% of participants, as classified by SAS scores, and 68.7% showed signs of depression based on SDS classification, suggesting a high burden of psychological distress.

**Table 3:** Physical activity levels, anxiety, and depression assessment in perimenopausal women (n = 387)

Item	Mean ($\overline{x}$) ± standard deviation (*s*)/[*M* (*Q1*, *Q3*)]
Total physical activity level (MET-min/w)	5301.48 ± 4575.24
Work-related (MET-min/w)	198 (0, 2199)
Daily transportation (MET-min/w)	894 (360, 1800)
Daily living (MET-min/w)	840 (281, 1954)
Leisure-time physical activity (MET-min/w)	396 (0, 990)
Sedentary time (min/w)	1598.00 ± 903.00
Sleep duration (min/w)	2773.00 ± 555.00
SAS	51.93 ± 10.43
SDS	59.78 ± 15.07

*MET* metabolic equivalent of task, *SAS* self-rating anxiety scale, *SDS* self-rating depression scale, *min/w* minutes per week, *M(Q1, Q3)* median (first quartile, third quartile)

### Correlations between quality of life and sleep, psychological, and physical activity variables

Significant correlations were observed between MENQOL scores and variables related to sleep, psychological status, and physical activity (Table 4). Total MENQOL scores were positively correlated with PSQI scores (r = 0.579, P < 0.01), indicating that poorer sleep quality was associated with a lower quality of life. A significant negative correlation was found with sleep duration (r = − 0.380, P < 0.01), suggesting that shorter sleep duration was linked to worse quality of life. Moderate positive correlations were also noted between MENQOL scores and both SAS (r = 0.096, P > 0.05) and SDS scores (r = 0.059, P > 0.05), reflecting a trend toward the negative impact of anxiety and depressive symptoms, although these associations were not statistically significant. Regarding physical activity, work-related activity (r = 0.144, P < 0.01) and total physical activity (r = 0.135, P < 0.01) were positively correlated with higher MENQOL scores, indicating a potential association between increased physical burden and reduced quality of life. In contrast, leisure-time physical activity showed no significant beneficial relationship with MENQOL scores.

**Table 4:** Correlation analysis between quality of life domains and related variables in perimenopausal women (n = 387) (r values, n = 387)

Variable	Total QoL score	Vasomotor symptoms mean score	Psychosocial status mean score	Physical status mean score	Sexual life mean score
BMI	0.055	0.073	0.029	0.084	− 0.068
Pittsburgh sleep quality index	0.579**	0.297**	0.494**	0.618**	0.235**
International physical activity level	0.135**	0.158**	0.127*	0.071	0.180**
Work-related activities	0.144**	0.170**	0.137**	0.08	0.176**
Daily transportation	0.037	− 0.024	0.114*	− 0.013	0.074
Daily living activities	0.095	0.129*	0.027	0.104*	0.055
Leisure-time physical activity	− 0.072	− 0.025	− 0.071	− 0.1	0.051
Sedentary time	− 0.049	− 0.127*	− 0.047	− 0.004	− 0.071
Sleeping time	− 0.380**	− 0.313**	− 0.348**	− 0.304**	− 0.320**
SAS	0.096	0.054	0.137**	0.115*	0.107*
SDS	0.059	0.125*	0.006	0.007	0.263**

*QoL* quality of life, *BMI* body mass index, *SAS* self-rating anxiety scale, *SDS* self-rating depression scale, *MET* metabolic equivalent of task

**P < 0.01; *P < 0.05

### Univariate factors associated with impaired quality of life

Univariate analysis revealed that age, residence, education, menopausal status, and comorbid conditions including hypertension, diabetes, coronary heart disease, and osteoporosis significantly influenced total MENQOL scores and its subdomains (P < 0.05) (Table 5). Older age was associated with higher symptom scores across all dimensions, particularly sexual and vasomotor symptoms. Women residing in rural areas and those with lower education levels reported worse quality of life. The presence of osteoporosis and cardiovascular diseases was consistently associated with higher MENQOL scores, suggesting substantial impairment in well-being.

**Table 5:** Univariate analysis of quality of life according to sociodemographic and clinical factors in perimenopausal women

Variable	Classification	n	Total QoL score	Vasomotor symptoms	Psychosocial status	Physical status	Sexual life
Age	40–45	88	42.90 ± 29.15	0.33 (0, 2.25)	1.72 ± 1.39	1.42 ± 1.08	1.60 ± 1.52
	46–50	186	48.55 ± 29.68	1 (0, 2.67)	1.82 ± 1.28	1.53 ± 1.00	2.24 ± 1.63
	51–55	85	64.89 ± 25.95	2.33 (1.00, 4.00)	2.37 ± 1.32	1.96 ± 0.88	3.31 ± 1.60
	56–60	28	58.71 ± 34.78	1.50 (0.08, 3.67)	2.20 ± 1.40	1.80 ± 1.18	2.81 ± 1.76
	F		9.719	9.334	4.714	5.192	17.458
	P		< 0.001	< 0.001	0.003	0.002	< 0.001
Place of residence	Rural	38	65.26 ± 33.23	2.00 (1.00, 3.75)	2.52 ± 1.49	1.96 ± 1.08	3.09 ± 1.71
	Town	48	52.29 ± 26.89	1.00 (0.33, 2.58)	2.09 ± 1.27	1.63 ± 1.00	2.26 ± 1.51
	City	301	49.75 ± 29.89	1.00 (0, 2.67)	1.85 ± 1.32	1.57 ± 1.02	2.30 ± 1.72
	F		4.56	3.602	4.697	2.39	3.811
	P		0.011	0.028	0.01	0.093	0.023
Education level	Primary school or below	23	73.70 ± 31.90	3.00 (1.00, 4.67)	3.04 ± 1.47	2.06 ± 1.01	1.81 (1.38, 3.25)
	Junior high school	55	59.55 ± 31.23	1.67 (0.67, 5.00)	2.22 ± 1.46	1.79 ± 0.98	1.75 (1.00, 2.38)
	High school or technical school	90	51.07 ± 28.86	1.83 (0.67, 3.67)	1.90 ± 1.27	1.51 ± 0.97	1.31 (0.73, 2.25)
	Associate degree	63	49.71 ± 25.87	2 (0, 3)	1.83 ± 1.10	1.60 ± 0.88	1.69 (1.00, 2.19)
	Bachelor’s degree	130	46.54 ± 30.92	0.33 (0, 1.67)	1.77 ± 1.34	1.57 ± 1.14	1.25 (0.69, 2.31)
	Master’s degree or above	26	46.85 ± 26.81	0.83 (0, 1.42)	1.75 ± 1.28	1.51 ± 0.95	1.34 (0.75, 2.02)
	F/H		4.329	46.632^2^	4.371	1.478	30.817^2^
	P		0.001	< 0.001	0.001	0.196	< 0.001
Occupation	Civil servant	5	39.20 ± 16.80	1.33 (0, 4.00)	1.34 ± 1.09	1.03 ± 0.67	2.60 ± 1.85
	Professional/technical staff	139	47.96 ± 31.08	0.67 (0, 2.00)	1.84 ± 1.36	1.60 ± 1.15	2.14 ± 1.71
	Clerical staff	73	47.34 ± 28.71	0.67 (0, 2.83)	1.80 ± 1.28	1.47 ± 0.95	2.14 ± 1.78
	Enterprise manager	11	55.27 ± 25.58	1.00 (0.33, 4.33)	2.35 ± 1.31	1.55 ± 0.61	2.91 ± 1.54
	Worker	40	59.30 ± 23.90	2.83 (2.08, 4.00)	2.03 ± 1.12	1.86 ± 0.80	2.33 ± 0.96
	Farmer	24	62.75 ± 31.91	2.34 (1.00, 3.92)	2.24 ± 1.37	1.86 ± 1.04	3.25 ± 1.61
	Self-employed	31	50.42 ± 30.24	1.00 (0, 2.67)	2.11 ± 1.43	1.52 ± 0.95	2.22 ± 1.77
	Retired	35	59.03 ± 31.91	3.00 (1.00, 4.00)	2.02 ± 1.45	1.78 ± 1.00	2.96 ± 1.74
	Unemployed	29	52.86 ± 33.52	1.33 (0.33, 2.50)	2.16 ± 1.48	1.58 ± 1.11	2.57 ± 2.03
	F		1.595	7.765	0.81	1.002	2.091
	P		0.125	< 0.001	0.594	0.434	0.036
Marital status	Unmarried	5	30.40 ± 13.39	1.67 (0, 2.33)	0.86 (0.64, 1.64)	0.94 (0.53, 1.41)	0.67 (0, 2.67)
	First marriage	363	52.13 ± 30.10	1.00 (0, 3.00)	1.86 (1.00, 2.71)	1.56 (0.81, 2.31)	2.00 (1.00, 3.67)
	Remarried	15	44.27 ± 30.80	1.33 (0, 2.67)	1.86 (0.71, 3.00)	0.81 (0.56, 1.00)	1.67 (1.00, 2.67)
	Divorced	4	56.75 ± 43.78	2.50 (0.58, 2.92)	2.29 (0.79, 4.96)	1.125 (0.75, 3.14)	1.50 (0, 3.75)
	F/H		1.198	0.496^2^	3.094^2^	4.04^2^	5.572^2^
	P		0.31	0.92	0.377	0.257	0.134
Monthly household income per capita	≤ 4999	111	54.58 ± 30.23	1.33 (0.33, 3.67)	2.07 ± 1.36	1.65 ± 0.97	2.55 ± 1.69
	5000–9999	165	49.62 ± 29.07	1.67 (0.17, 2.67)	1.79 ± 1.26	1.58 ± 1.02	2.27 ± 1.63
	10,000–19,999	83	51.52 ± 29.67	0.67 (0, 2.33)	2.07 ± 1.35	1.65 ± 1.05	2.32 ± 1.78
	≥ 20,000	28	51.57 ± 37.56	1.00 (0, 2.67)	2.03 ± 1.66	1.62 ± 1.25	2.36 ± 2.00
	F		0.595	3.709	1.319	0.157	0.607
	P		0.619	0.012	0.268	0.925	0.611
Menopausal status	No	271	47.40 ± 29.74	1.00 (0, 2.67)	1.80 ± 1.31	1.52 ± 1.04	2.05 ± 1.63
	Yes	116	61.38 ± 28.94	2.33 (0.67, 3.58)	2.29 ± 1.34	1.84 ± 0.97	3.11 ± 1.65
	F		18.232	16.67	11.074	8.205	34.478
	P		< 0.001	< 0.001	0.001	0.004	< 0.001
Age of menarche	≤ 12	91	54.03 ± 27.54	1.33 (0, 2.67)	1.96 ± 1.24	1.75 ± 0.98	2.33 (1.33, 3.67)
	13–16	276	51.07 ± 31.17	1.00 (0, 3.00)	1.95 ± 1.39	1.58 ± 1.04	2 (1, 4)
	≥ 17	20	47.75 ± 27.67	1.00 (0, 2.92)	1.83 ± 1.16	1.54 ± 1.08	1.67 (0.75, 2.83)
	F/H		0.501	0.035	0.081	1.017	2.138^2^
	P		0.606	0.966	0.922	0.363	0.343
Number of childbirths	0	13	55.00 ± 30.89	2.00 (0, 2.67)	1.79 ± 1.13	1.79 ± 1.13	2.41 ± 1.75
	1	262	52.40 ± 29.99	1.33 (0, 3.00)	1.61 ± 1.00	1.61 ± 1.00	2.52 ± 1.74
	2	106	48.88 ± 30.40	1.00 (0, 2.42)	1.59 ± 1.05	1.59 ± 1.05	2.00 ± 1.59
	≥ 3	5	45.20 ± 24.93	1.67 (0.50, 3.33)	1.45 ± 0.82	1.45 ± 0.82	2.00 ± 1.55
	F		0.478	1.352	0.326	0.19	2.404
	P		0.698	0.257	0.806	0.903	0.067
Number of abortions	0	120	47.86 ± 28.93	1.00 (0, 2.67)	1.79 ± 1.38	1.52 ± 1.00	2.14 ± 1.67
	1	143	52.17 ± 31.71	1.33 (0, 3.00)	1.99 ± 1.37	1.62 ± 1.06	2.38 ± 1.76
	2	89	51.76 ± 28.15	1.00 (0.33, 2.83)	1.93 ± 1.22	1.59 ± 0.99	2.56 ± 1.65
	≥ 3	35	61.57 ± 31.45	1.00 (0, 3.17)	2.33 ± 1.35	2.00 ± 1.06	2.63 ± 1.74
	F		1.924	0.432	1.556	1.995	1.333
	P		0.125	0.73	0.2	0.114	0.263
Hypertension	No	358	50.56 ± 30.45	1.00 (0, 2.67)	1.91 ± 1.35	1.50 (0.75, 2.25)	2.32 ± 1.72
	Yes	29	64.28 ± 23.09	2.33 (1.00, 3.50)	2.42 ± 1.07	1.94 (1.34, 2.59)	3.01 ± 1.43
	F/Z		5.613	5.917	4.011	− 2.314^1^	4.495
	P		0.018	0.015	0.046	0.021	0.035
Diabetes	No	375	50.75 ± 30.15	1.00 (0, 2.67)	1.92 ± 1.34	1.00 (0, 2.67)	2.00 (1.00, 3.67)
	Yes	12	77.75 ± 15.27	3.00 (2.08, 3.92)	2.81 ± 0.84	3(2.08,3.92)	3.00 (3.00, 3.33)
	F/Z		− 3.44^1^	5.917	4.011	− 3.354^1^	− 2.094^1^
	P		0.001	0.015	0.046	0.001	0.036
Coronary heart disease	No	382	51.21 ± 30.10	1.64 ± 1.64	1.93 ± 1.33	1.61 ± 1.03	2.35 ± 1.70
	Yes	5	80.4 ± 19.26	2.73 ± 1.64	3.43 ± 1.15	2.26 ± 0.71	4.00 ± 1.25
	F		4.667	2.175	6.283	2.002	4.68
	P		0.031	0.141	0.013	0.158	0.031
Osteoporosis	No	378	50.80 ± 29.62	1.61 ± 1.61	1.91 ± 1.31	1.60 ± 1.02	2.33 ± 1.69
	Yes	9	84.78 ± 35.58	3.52 ± 1.83	3.54 ± 1.68	2.35 ± 1.05	3.93 ± 1.68
	F		11.463	12.16	13.448	4.785	7.82
	P		0.001	0.001	< 0.001	0.029	0.005

QoL quality of life, *BMI* body mass index, *F* F-statistic, *H* Kruskal–Wallis H-statistic, *Z* Z-statistic, *P* P-value, *n* number of participants

^1^Z-value; ^2^H-value

### Independent predictors of reduced quality of life identified by multivariate analysis

Multivariate linear regression identified several independent predictors of total MENQOL scores (Table 6). Poorer quality of life was significantly associated with older age (β = 0.107, P = 0.011), lower sleep quality (β = 0.514, P < 0.001), shorter sleep duration (β = –0.145, P = 0.001), higher levels of work-related physical activity (β = 0.090, P = 0.026), and the presence of comorbid coronary heart disease (β = 0.106, P = 0.006) and osteoporosis (β = 0.115, P = 0.003). These findings highlight the contribution of both modifiable lifestyle factors and chronic health conditions to reduced quality of life during perimenopause.

**Table 6:** Multiple linear regression analysis of quality of life in perimenopausal women

Dependent variable	Independent variable	Regression coefficient	Standard error	Standardized regression coefficient	t-value	P-value
Total quality of life score	Constant	43.413	9.385	–	4.626	< 0.001
	Age	3.773	1.479	0.107	2.551	0.011
	Osteoporosis	22.795	7.715	0.115	2.955	0.003
	Coronary heart disease	28.158	10.277	0.106	2.74	0.006
	Pittsburgh sleep quality index	2.619	0.207	0.514	12.67	< 0.001
	Sleep duration	− 0.008	0.002	− 0.145	− 3.394	0.001
	Physical activity at work	0.001	0	0.09	2.238	0.026
Vasomotor symptoms	Constant	3.574	0.576	–	6.209	< 0.001
	Education level	− 0.279	0.056	− 0.235	− 4.971	< 0.001
	Age	0.191	0.092	0.099	2.076	0.039
	Osteoporosis	1.452	0.478	0.134	3.036	0.003
	Diabetes	1.042	0.419	0.111	2.487	0.013
	SDS	0.017	0.005	0.159	3.429	0.001
	Pittsburgh sleep quality index	0.069	0.013	0.247	5.176	< 0.001
	Sleep duration	0	0	− 0.12	− 2.457	0.014
Psychosocial status	Constant	2.437	0.355	–	6.867	< 0.001
	Education level	-0.108	0.042	− 0.111	− 2.581	0.01
	Osteoporosis	1.228	0.371	0.14	3.313	0.001
	Coronary heart disease	1.46	0.494	0.124	2.956	0.003
	Pittsburgh sleep quality index	0.098	0.01	0.432	9.826	< 0.001
	Sleep duration	0	0	− 0.166	− 3.684	< 0.001
Physical status	Constant	1.109	0.256	–	4.326	< 0.001
	Pittsburgh sleep quality index	0.102	0.007	0.584	14.104	< 0.001
	Sleep duration	0	0	− 0.114	− 2.732	0.007
	Physical activity in daily life	5.36	0	0.093	2.338	0.02
Sexual life	Constant	3.134	0.609	–	5.143	< 0.001
	Sleep duration	0	0	− 0.11	− 2.327	0.02
	Menopausal status	0.598	0.182	0.161	3.285	0.001
	SAS	0.034	0.013	0.207	2.592	0.01
	SDS	0.055	0.009	0.482	5.995	< 0.001
	Pittsburgh sleep quality index	0.061	0.013	0.21	4.574	< 0.001
	Age	0.361	0.101	0.18	3.566	< 0.001
	Physical activity at work	5.89	0	0.111	2.553	0.011
	Coronary heart disease	1.341	0.64	0.089	2.094	0.037

The total score for perimenopausal quality of life was R^2^ = 0.436, adjusted R^2^ = 0.426, F = 41.746, *P* < 0.001; vasomotor symptoms were R^2^ = 0.271, adjusted R^2^ = 0.257, F = 19.972, *P* < 0.001; psychosocial status was R^2^ = 0.332, adjusted R^2^ = 0.323, F = 37.695, *P* < 0.001; physical status was R^2^ = 0.404, adjusted R^2^ = 0.400, F = 86.194, *P* < 0.001; sexual life was R^2^ = 0.322, adjusted R^2^ = 0.308, F = 22.416, *P* < 0.001

*QoL* quality of life, *SAS* self-rating anxiety scale, *SDS* self-rating depression scale, *R*^*2*^ coefficient of determination, *F* F-statistic, *P* P-value

In the vasomotor domain, more severe symptoms were significantly associated with lower education levels, older age, comorbid osteoporosis and diabetes, and higher SDS scores, indicating that both sociodemographic and psychological factors contribute to vasomotor complaints. In the psychosocial domain, poor sleep quality, shorter sleep duration, coronary heart disease, and lower educational attainment were identified as significant predictors, reflecting the interplay between physical health, mental well-being, and social context.

In the physical domain, sleep quality emerged as the strongest predictor (β = 0.584, P < 0.001), followed by sleep duration and intensity of daily life physical activity, suggesting that physical burden and sleep disturbances exacerbate somatic complaints. In the sexual domain, impaired quality of life was independently associated with menopausal status, higher anxiety and depression scores (SAS and SDS), coronary heart disease, poor sleep quality, and reduced sleep duration. These results emphasize the complex interaction between physiological changes, comorbidities, and mental health in determining sexual well-being during the menopausal transition.

Model assumptions were tested and confirmed to be appropriate. P–P plots demonstrated that residuals approximated a normal distribution, and Durbin–Watson statistics ranged from 1.654 to 1.858, indicating independence of residuals. Variance inflation factors (VIFs) were between 1.008 and 3.589, showing no significant multicollinearity among independent variables. The adjusted R2 values ranged from 0.257 for vasomotor symptoms to 0.426 for total MENQOL scores, reflecting moderate explanatory power across domains. These findings underscore the need for integrated interventions targeting sleep quality, mental health, and chronic disease management to improve the quality of life among perimenopausal women.

## Discussion

This study revealed that perimenopausal women exhibited elevated MENQOL scores across all dimensions, particularly in psychosocial and sexual domains, suggesting a generally reduced quality of life in these areas. The mean age of participants was 47.94 ± 5.30 years, with 62.1% aged 46–55 years, corroborating prior evidence that this age range corresponds to the peak period of perimenopausal symptomatology. Moreover, a large proportion of participants experienced poor sleep quality, with an average PSQI score of 9.97 ± 5.89 and 70.5% scoring ≥ 7, indicative of disturbed sleep.

These findings align with national data indicating a high prevalence of perimenopausal syndrome in China, reportedly affecting up to 70% of women, with common symptoms including vasomotor instability, musculoskeletal pain, and genitourinary changes [32]. Consistent with our results, previous literature has highlighted poor sleep quality as a major contributor to perimenopausal symptom burden, with the National Institutes of Health also underscoring sleep disorders as a key health concern during this transition [33, 34].

The high scores in the psychosocial and sexual life domains further reflect significant emotional and interpersonal challenges. In our study, 58.9% of women exhibited symptoms of anxiety (SAS score > 50), and 68.7% demonstrated symptoms of depression (SDS score > 53). While specific psychiatric diagnostic instruments were not applied, these elevated symptom scores suggest considerable psychological distress. In parallel, regional studies from China report sexual dysfunction rates nearing 48.7% among perimenopausal women [35], often attributable to estrogen decline and resultant vaginal atrophy, dryness, and dyspareunia [36]. International data echo this trend, with studies from Italy indicating that up to 72.6% of women report sexual dysfunction during the menopausal transition [37].

Multivariate regression analyses identified age, sleep quality, sleep duration, comorbid coronary heart disease, osteoporosis, and work-related physical activity as independent predictors of total MENQOL score. Notably, poorer sleep quality (higher PSQI scores) and shorter sleep duration were significantly associated with worse quality of life, consistent with previous research highlighting the central role of sleep in mediating menopausal symptom burden and quality of life [38].

Our findings also suggest that age is a relevant factor influencing total quality of life, vasomotor symptoms, and sexual life domains. As women transition through perimenopause, declining ovarian function and hormonal changes are known to contribute to symptoms such as hot flashes and genitourinary syndrome, thereby reducing quality of life. This is consistent with prior reports that the average menopausal age in China is 48–52 years and that earlier menopause is increasingly common [39].

Chronic conditions such as coronary heart disease and osteoporosis emerged as additional independent risk factors. These findings are supported by the pathophysiological impact of estrogen deficiency, which contributes to lipid profile deterioration, vascular changes, and bone demineralization [40]. The identification of these diseases as predictors highlights the importance of early screening and management of chronic comorbidities during the perimenopausal transition.

In terms of physical activity, regression analysis showed that higher levels of work-related physical activity were significantly associated with poorer quality of life (P = 0.026). Although the study did not separately assess recreational or exercise-specific activity in the multivariate model, the observed correlation between work-related activity and reduced quality of life suggests that the type and context of physical exertion may differentially influence outcomes. Previous studies have shown that structured exercise, such as walking or yoga, may alleviate vasomotor and psychological symptoms [41], while work-related physical activity, often obligatory and fatiguing, may not confer similar benefits.

A deeper analysis uncovered key factors of physical activity that are associated with perimenopausal quality of life. Higher levels of overall physical activity, work-related physical activity, and daily living physical activity did not lead to improved quality of life; instead, they were associated with relatively lower quality of life. Conversely, increased levels of exercise and recreational activities were associated with better quality of life. These findings suggest that appropriate levels of physical activity, particularly exercise and recreational activities, are critical for improving quality of life in perimenopausal women.

Lastly, we found that depressive symptoms, as measured by SDS, were found to be significantly associated with reduced quality of life in specific domains. Correlation analysis revealed that SDS scores were positively correlated with vasomotor symptoms and sexual symptoms, while multiple linear regression indicated that SDS remained an independent predictor of sexual life quality. These findings suggest that higher levels of depressive symptoms were strongly associated with greater sexual dysfunction in perimenopausal women. Although no significant regression association was observed between SDS and psychosocial status in the final model, the correlation analysis indicated a modest but positive relationship between SDS and psychosocial domain scores. This indicates that depressive symptoms may contribute to psychological distress during the perimenopausal period, potentially exacerbating emotional instability and interpersonal difficulties [42]. These observations underscore the importance of identifying and managing depressive symptoms in perimenopausal women to improve overall quality of life, particularly in aspects related to emotional and sexual well-being.

Although our current study provides insights into the quality of life among perimenopausal women, highlighting the influence of age, sleep quality, physical activity, and comorbidities. However, several limitations should be noted. First, the study was conducted at a single center using convenience sampling, which may introduce selection bias and limit the generalizability of the findings to other populations or regions. Second, although the SAS and SDS were used to assess emotional status, the primary focus of this study was not the diagnosis or clinical evaluation of mental disorders. While the results showed that higher SAS and SDS scores were associated with poorer quality of life in multiple domains, the analysis did not include detailed stratification or separate modeling of depression as an independent predictor. Third, this study relied on self-reported questionnaires, which may be subject to recall bias and social desirability bias, potentially affecting the accuracy of responses related to sleep, mood, and physical activity. Fourth, the cross-sectional design precludes any inference of causality between influencing factors (e.g., sleep quality, physical activity, comorbidities) and quality of life. Future studies should aim to adopt multicenter, prospective designs with larger and more diverse samples. Incorporating clinical diagnostic tools for emotional and psychological assessments and objectively measured physical activity data may help strengthen the reliability and depth of future findings.

## Conclusion

This study highlights the multidimensional impact of various factors on the quality of life in perimenopausal women. Poor sleep quality, shorter sleep duration, and the presence of chronic conditions such as coronary heart disease and osteoporosis were independently associated with higher MENQOL scores, indicating poorer quality of life. Furthermore, older age and increased physical activity at work were also identified as significant predictors of reduced well-being. These findings underscore the need for comprehensive management strategies that address not only physical health and menopausal symptoms but also sleep and mental health support to improve the overall quality of life in this population. Future research should incorporate larger, more diverse populations and longitudinal designs to further validate and expand upon these observations.

## Data Availability

The datasets used and analysed during the current study are available from the corresponding author on reasonable request.
